# A closer look at how the dispersive liquid–liquid microextraction method works. Investigation of the effect of solvent mixture composition on the quality and stability of the cloudy state

**DOI:** 10.3389/fchem.2024.1383445

**Published:** 2024-06-11

**Authors:** Serhii Zaruba, Michaela Ovšonková, Patrycja Makoś-Chełstowska, Vasil Andruch

**Affiliations:** ^1^ Department of Analytical Chemistry, Institute of Chemistry, Faculty of Science, Pavol Jozef Šafárik University in Košice, Košice, Slovakia; ^2^ Department of Process Engineering and Chemical Technology, Faculty of Chemistry, Gdańsk University of Technology, Gdańsk, Poland

**Keywords:** DLLME, dispersive liquid-liquid microextraction, dispersion degree, dispersion stability, cloudy state, LPME, liquid-phase microextraction

## Abstract

The dispersive liquid–liquid microextraction (DLLME) is one of the most popular miniaturized extraction procedures. In this paper, the degree of dispersion and dispersion stability were studied with the aim to assess the correlations of these parameters with efficiency for the selected analytical application. The dependence between the degree of dispersion (cloudy state quality) and its stability obtained by various emulsification procedures, such as solvent-assisted emulsification (using various dispersive solvents) and mechanical emulsification (using auxiliary energies), is investigated and discussed. It was found out that the degree of dispersion depends on the type of emulsification procedure and decreases in the series: solvent-assisted (SA-) = ultrasound-assisted (UA-) > air-assisted (AA-) > vortex-assisted (VA-) emulsification. The emulsion stability depends on the degree of dispersion and there were 1810 and 2070 s for the most effective emulsification procedures, such us solvent-assisted and ultrasound-assisted emulsification, respectively. A comparison between the sensitivity of the analytical methods (using spectrophotometric determination of the anionic surfactants) and the degree of dispersion have been made. The sensitivity of the methods was ranked as follows: DLLME > UA-LLME > VA-LLME > AA-LLME.

## Graphical Abstract

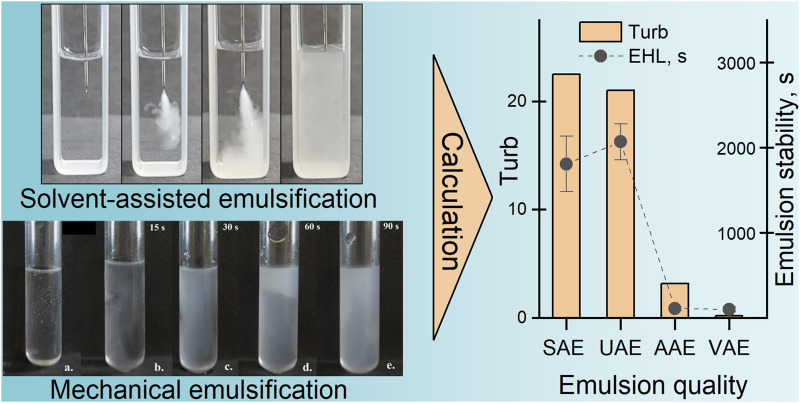

## Highlights

• The degree of dispersion and emulsion stability depend on emulsification procedure.
• In microextraction methods the emulsion quality affect on its stability.
• The quality of the dispersion depends on the extraction-to-dispersive solvent ratio.
• UAE provides the best emulsion quality among mechanical emulsification techniques.
• The sensitivity of microextraction methods depend on the degree of emulsification.

## 1 Introduction

At the beginning of this century, Rezaee et al. (Berijani et al., 2006; Rezaee et al., 2006) introduced a new microextraction technique called dispersive liquid–liquid microextraction (DLLME), which attracted the attention of researchers in the field of analytical chemistry (Campillo et al., 2017). The procedure is based on a ternary-component system in which a suitable mixture of extraction solvent and dispersive solvent is rapidly injected into an aqueous sample resulting in the formation of a cloudy state. The dispersive solvent should contribute to the formation of fine droplets of the extraction solvent in the aqueous sample, which leads to an increase in the contact surface between the two phases, thus enabling better and faster mass transfer. However, the role of dispersive solvent has been studied to a lesser extent (Kagaya et al., 2010; Kocúrová et al., 2013); therefore, a full understanding of its function is still an open question.

Among the main advantages of DLLME are its ease of operation, rapidity, high recovery and high enrichment factor. To ensure the high extraction efficiency of DLLME, the droplets of the extraction solvent in the aqueous solution should be as small as possible. Numerous fine droplets provide a large contact area between the aqueous and organic phases, consequently improving the mass transfer process. The cloudy solution (emulsion) should be stable during the extraction process. Unstable systems lead to coalescence, Ostwald ripening or flocculation, that is, the merging of fine droplets of the extraction solvent into much larger ones, thereby reducing the extraction efficiency (Moradi et al., 2011; Alade et al., 2021).

In addition to using a dispersive solvent, the dispersion of the extraction solvent in the aqueous sample can also be achieved using various auxiliary energies, such as ultrasound in ultrasound-assisted liquid–liquid microextraction (UALLME) (Regueiro et al., 2008), a vortex in vortex-assisted liquid–liquid microextraction (VALLME) (Yiantzi et al., 2010), air mixing in air-assisted liquid–liquid microextraction (AALLME) (Farajzadeh and Mogaddam, 2012) or by combination of these two approaches (Šandrejová et al., 2016; Campillo et al., 2017). Although DLLME and its modifications have been known for almost 20 years and numerous papers have focused on its application for the determination of various analytes in various samples, to our knowledge there are only a handful of publications in which the authors also studied the formation and stability of the cloudy state (Liao et al., 2011; Kocúrová et al., 2013). Most often, authors limit themselves to a statement that cloudy state is formed and focus mainly on the development of analytical procedure.

Therefore, the aim of this paper is to study the degree of dispersion (cloudy state quality) and its stability obtained by various emulsification procedures, such as solvent-assisted emulsification (using various dispersive solvents) and mechanical emulsification (using auxiliary energies). The study also aimed to identify potential relationships between the quality and stability of the cloudy state and the effectiveness of the chosen analytical application, which involved employing spectrophotometry for the determination of anionic surfactants as a model procedure.

## 2 Materials and methods

### 2.1 Chemicals and reagents

All chemicals and reagents used in this study were of analytical grade unless otherwise stated. Organic solvents, such as toluene, acetonitrile, acetone and methanol, were purchased from Centralchem (Slovakia), and ethanol (96%) was obtained from ITES (Slovakia). The 0.01 mol L^–1^ stock solution of anionic surfactant was prepared by dissolving an appropriate amount of sodium dodecylbenzenesulfonate (DBS) (technical grade, Sigma-Aldrich, France) in 100 mL of water. The working solutions of Na-DBS were prepared by appropriate dilution of the stock solution. The 2.5 mmol L^–1^ solution of cationic dye was prepared by dissolving 0.142 g of solid Crystal Violette (CV) nonahydrate (LACHEMA) in 100 mL of water. The 1.25 mol L^–1^ Na_2_SO_4_ was prepared by dissolving 40.28 g of Na_2_SO_4_·10 H_2_O in 100 mL of water. An acetate buffer solution of pH 5.0 was prepared by dissolving 0.957 g of sodium acetate trihydrate in 80 mL of water and adding 0.190 g of glacial acetic acid. Then the pH value was adjusted with HCl or NaOH solution and the volume was made up to 100 mL by water. Ultrapure water from WATEK (Czech Republic) was used throughout the work (18.2 MΩ cm).

### 2.2 Apparatus

A SPECORD S 600 UV–vis spectrophotometer (Analytik Jena, Germany) with a matched glass cell of 2 mm path length and a matched quartz cell of 10 mm path length were used for the absorbance measurements. A UCI-150 ultrasonic water bath (RAYPA, Spain) equipped with a high frequency generator (325 W of power and 35 kHz of frequency) and a VM-3000MD vortex mixer (Medline Scientific, United Kingdom) were used to assist the dispersion of toluene in the water phase and extraction process. Centrifugation was carried out using a CN-2060 centrifuge (MRC, Israel). A transmitting light optical microscope with a 20x/0.45 and 40x/0.65 working distance lens (LAB 40 Series Optical Microscope, OPTA-TECH, Poland) was used to measure the diameters of the emulsion droplets.

### 2.3 Emulsification procedures

#### 2.3.1 Procedure of solvent-assisted emulsification (SAE)

**FIGURE 1:**
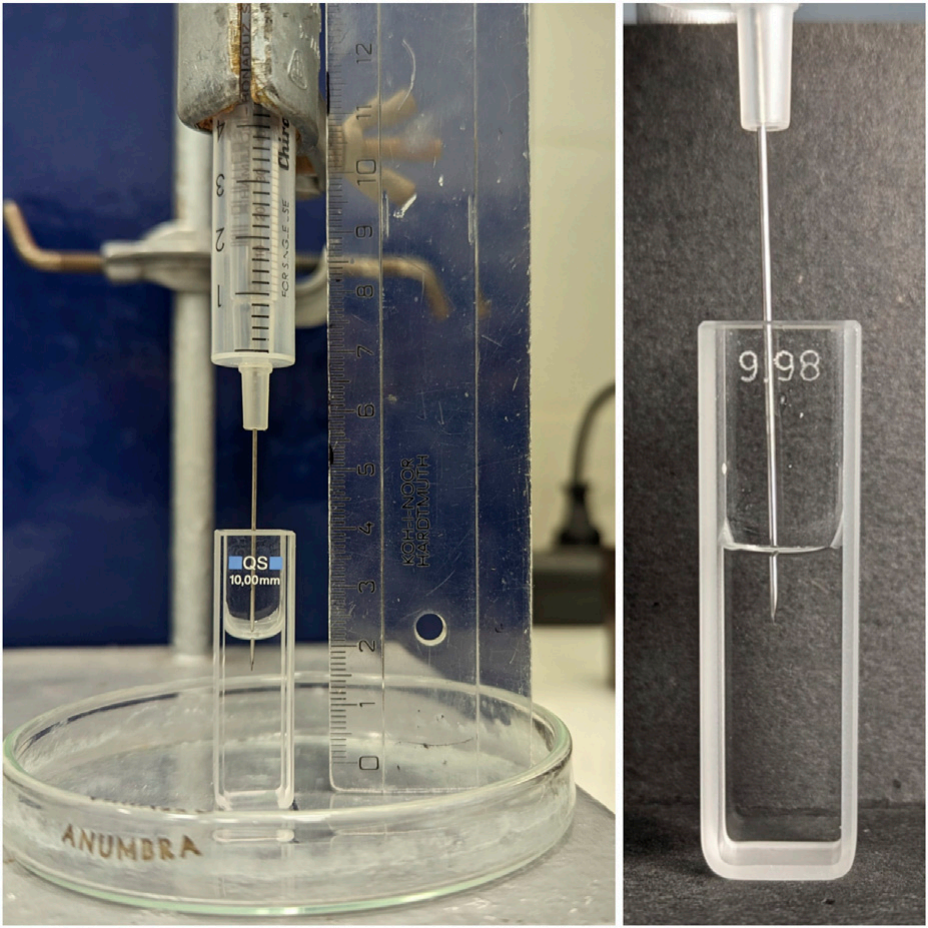
Image of the experimental device for holding the microsyringe.

**FIGURE 2:**
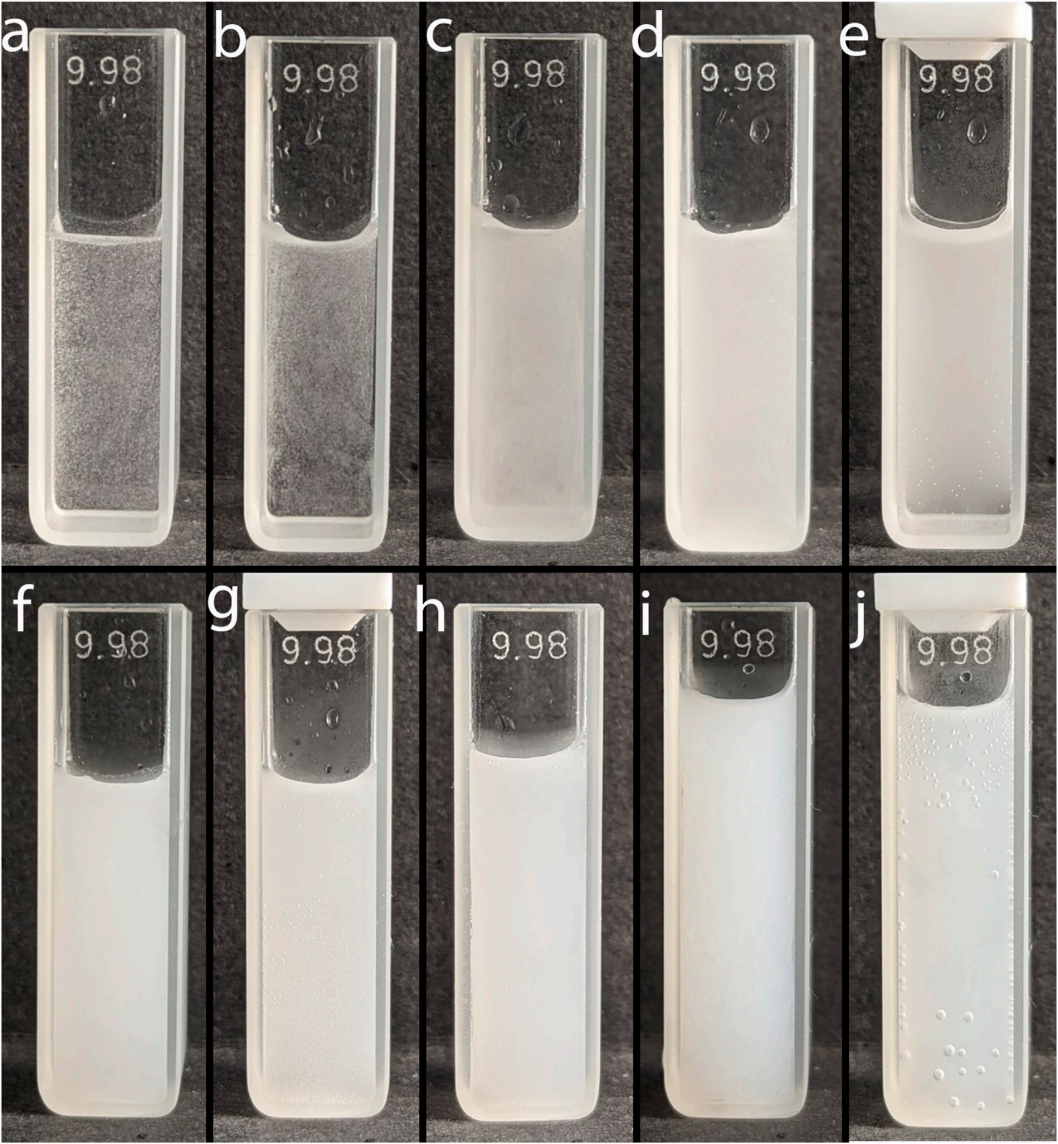
Photo of emulsions of toluene (8.3 μL) in water (2.5 mL) obtained by adding a toluene-methanol mixture at different ratios. T:M ratios: **(A)** 1:1 (0 s), **(B)** 1:2 (0 s), **(C)** 1:5 (0 s), **(D)** 1:10 (0 s), **(E)** 1:10 (600 s), **(F)** 1:25 (0 s), **(G)** 1:25 (600 s), **(H)** 1:50 (0 s), **(I)** 1:100 (0 s), **(J)** 1:100 (600 s).

This emulsification procedure is based on the addition of a mixture of extraction and dispersive solvent to the aqueous sample and corresponds to the conventional DLLME procedure in terms of analytical chemistry. Various solvent mixtures with different volume ratios of extraction and dispersive solvent (1:1, 1:2, 1:5, 1:10, 1:25, 1:50 and 1:100) were prepared just before the experiment. A 10 mm quartz cuvette with a total volume of approximately 3.5 mL containing 2.5 mL of water was placed on a Petri dish. The microsyringe was fixed (Figure 1) above the cuvette using a laboratory holder. Fixing the microsyringe ensured a constant position of the needle (in the center of the cuvette approximately 7 mm below the surface) as well as a constant force of injection of the solvent mixture into the water. Then a certain volume of a pre-prepared mixture of solvents (17, 25, 50, 90, 220, 420 and 840 μL) always containing the same volume of toluene, 8.3 μL, was rapidly injected into the aqueous phase using a chromatographic microsyringe (in the case of a volume of 840 μL, an automatic pipette was used). This resulted in the formation of a cloudy state (emulsion). Immediately after the injection of the solvent mixture, the cuvette was closed with a cap and gently shaken by hand to distribute the formed emulsion throughout the whole volume (Figure 2). For emulsions with low turbidity values, the cuvette was placed into the spectrophotometer, and absorbance measurements were started in kinetic mode at 600 nm at 3-s intervals over a period of 300 s for toluene:dispersive solvent ratio from 1:1 to 1:10, and for 2400 s at 30-s intervals for the 1:10 ratio of toluene with acetone or methanol. In the case of high turbidity values (a solvents ratio of more than 1:10), 250 μL of the emulsion prepared in 10 mm cuvettes was transferred to 2 mm cuvette and measurements were carried out in batch mode (a new batch of the same emulsion was taken for each measurement) at times 0, 5, 10, 20, 30 and 40 min starting from the preparation of the emulsion in a 10 mm cuvette.

Turbidity was calculated from the measured absorbance by the equation (Melik and Fogler, 1983; Aizawa, 2014):

$$Turb=\frac{\text{Abs}\times 2.3}{l}$$ (1)

where, Turb is the turbidity of emulsion, Abs is the absorbance measured at 600 nm, *l* is the path-length in cm.

The emulsion particle size was measured by optical microscopy using a Delta Optical ME 1000 light microscope (objective lenses ×20/0.45 and 160/0.17) interfaced with a computer. For each test, a drop of the emulsion sample was carefully placed on a microscope slide and transferred to the microscope. Then, images of the emulsion particles were recorded. The particle sizes were determined using Clemex Vision software. The diameters were measured based on at least 128 droplets (with 95% accuracy in the description). The asymmetric diameter of the emulsion droplets (d_a_, µm) was calculated using the equation:

$$d_{a}=\frac{\sum_{i=1}^{n}n_{i}∙d_{i}}{\sum_{i=1}^{n}n_{i}}$$ (2)

where d_i_, diameter of the droplet (µm); n_i_, number of elements with diameter d_i_ (−).

The average diameters of the particles in the emulsions were estimated using the Sauter mean volume diameter (d_32_), given by the equation:

$$d_{32}=\frac{\sum_{i=1}^{n}n_{i}∙d_{i}^{3}}{\sum_{i=1}^{n}n_{i}∙d_{i}^{2}}$$ (3)

#### 2.3.2 Procedures of mechanical emulsification

##### 2.3.2.1 Ultrasound-assisted emulsification (UAE)

This emulsification procedure is based on the application of ultrasound energy and corresponds to the UALLME procedure in terms of analytical chemistry. First, 2.5 mL of water was put in a glass tube (with an inner diameter of 10 mm, a length of 110 mm and a total volume of about 7.8 mL). Then, 8.3 μL of toluene was carefully placed on the water’s surface using a chromatographic syringe. The tube was tightly plugged with a PE cap and vigorously shaken by hand 20 times to break the thin film of extraction solvent on the surface. The tube was then immediately placed into an ultrasonic water bath and sonicated for a set time (0.5, 1, 1.5, 2, 3, 5 and 10 min) and a cloudy state was formed. Afterward, the tube was taken from the ultrasonic water bath and gently shaken to distribute the formed emulsion throughout the whole volume. Then 250 μL of the emulsion was transferred to a 2 mm cuvette and measurements at 600 nm were carried out in batch mode (a new batch of the same emulsion was taken for each measurement) at times 0, 5, 10, 20, 30 and 40 min, starting from the preparation of the emulsion, in a 10 mm cuvette.

##### 2.3.2.2 Vortex-assisted emulsification (VAE)

This emulsification procedure is based on the application of vortex mixing and corresponds to the VALLME procedure in terms of analytical chemistry. First, 2.5 mL of water was put in a glass tube (with an inner diameter of 10 mm, a length of 110 mm and a total volume of about 7.8 mL). Then, 8.3 μL of toluene was carefully placed on the water’s surface using a chromatographic syringe. The tube was tightly plugged with a PE cap. Then the mixture was stirred with vortex mixer for a set time (15, 30, 45, 60 and 90 s) at 3000 rpm. Afterward, the mixture was immediately transferred into a 10 mm cuvette and absorbance measurements at 600 nm were performed in kinetic mode at 3-s intervals over a period of 300 s.

##### 2.3.2.3 Air-assisted emulsification (AAE)

This emulsification procedure is based on mixing by repeated sucking and injecting of the mixture of aqueous sample and extraction solvent with a syringe and corresponds to the AALLME procedure in terms of analytical chemistry. First, 2.5 mL of water was put in a 10 mm quartz cuvette (total volume about 3.5 mL). Then, 8.3 μL of toluene was carefully placed on the water’s surface using a chromatographic syringe. Then the mixture was rapidly aspirated into a 5 mL PE syringe and then pushed out into a cuvette several times (3, 5, 10, 15, 20 times). Afterward, the mixture was immediately transferred to a 10 mm cuvette and absorbance measurements at 600 nm were made in kinetic mode at 3-s intervals over a period of 300 s.

### 2.4 Kinetic measurements and calculations

Emulsion sedimentation may follow from a zero-order (in case of unhindered settling) to a first- or mixed- (combined zero and first) order rate law reaction (Aizawa, 2014; Bol et al., 2021). Our preliminary investigations shown that in most cases the coefficients of determination of linearized graphs (*R* ^2^) were higher with the use of the first-order rate law compared to the zero-order. Therefore, for further kinetic calculations, we considered a first order reaction, which is described by following the mathematical formulas: reaction rate (4), half-life of the compound (emulsion half-life (EHL) in our case) (5), coordinates for kinetic curve linearization (6):

$$v=k\left[A\right]$$ (4)

$$t_{1/2}=\frac{\ln2}{k}$$ (5)

$$\ln\left[A\right]\;vs\;t$$ (6)

where, *k* is the reaction rate constant, [A] means the concentration of the compound (or Turbidity in our case), *t* is the time in seconds.

Graphs of turbidity vs. time and a linearized graph of ln (Turb) vs. time were plotted. The reaction rate constant (*k*) was calculated from slope of the straight-line:

$$k=‐\left(\text{slope}\;\text{of}\;\text{straight}‐\text{line}\right)$$ (7)

The straight-line was constructed through points that closely corresponded to the straight segment of the kinetic curve between the phases “dead” or “lag time phase” and the “holdup phase” close to the main phase boundary (Aizawa, 2014; Bol et al., 2021). Examples of emulsion sedimentation kinetic curves and their linearized versions are shown in Supplementary Material, Supplementary Figure S1.

### 2.5 General procedure for determination of anionic surfactants

The results obtained during the study of cloudy state formation were used to develop an analytical procedure for spectrophotometric determination of anionic surfactants as a model analyte. The effort was to follow as much as possible the extraction procedures used in the kinetic study, but in some cases their subtle modification was necessary.

The initial experimental conditions for the formation of the DBS-CV ion associate were taken from work (Motomizu et al., 1982), checked and adjusted to the microextraction conditions (The effect of variables are shown in Supplementary Material, Supplementary Figure S2). The procedure was designed to take into account the change in the volume of the extraction solvent from milliliter to microliter level. Briefly, 0.8 mL of 1.25 mol L^–1^ Na_2_SO_4_, 0.1 mL of acetic buffer solution with pH 5, 1 mL of sample solution or 0.14, 0.28, 0.56, 0.80 and 1.4 mL of 0.025 mmol L^–1^ Na-DBS, were placed into 15 mL PE centrifugal test tubes with a tightly screwed cap. After each reagent was added, the mixture was gently shaken. The total volume was made up to the 2.3 mL with water. Then 0.2 mL of 2.5 mmol L^–1^ CV was added and the mixture was vigorously mixed. The samples prepared in this way were then subjected to the various microextraction procedures, namely, conventional DLLME, UALLME, VALLME, AALLME and LPME (See Supplementary Material for experimental details, section *“Procedures for microextraction surfactant determination”*).

## 3 Results and discussion

**FIGURE 3:**
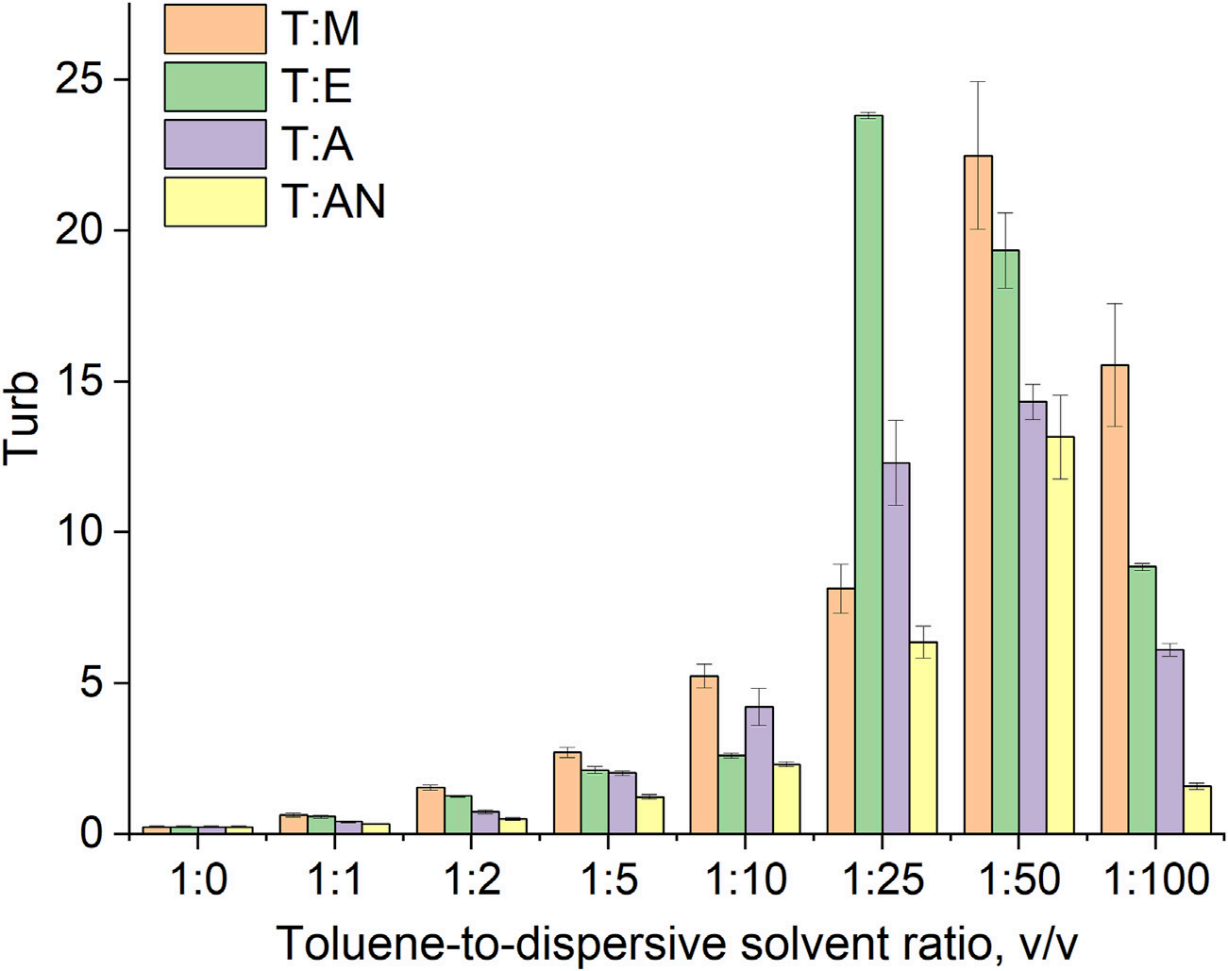
Dependence of system turbidity on the type of dispersive solvent used and the toluene-to-dispersive solvent ratio.

Among the important parameters that determine efficiency of the mass transfer in the DLLME method are the formation of numerous fine droplets of the extraction solvent dispersed throughout the aqueous phase and the stability of the emulsion formed. Therefore, the turbidity of such emulsions (Figure 3) as well as the diameters of the extraction solvent droplets were measured, and emulsion half-life (EHL) values were subsequently calculated (Sjöblom et al., 2013).

### 3.1 Study of the solvent-assisted emulsification

Toluene was used as a model extraction solvent due to its low solubility in the water, and good ability to extract of ion associate of CV-DBS (model analytical system). The volume of 8.3 µL of toluene was chosen mainly because of the limitations of the apparatus: the limited volume of the cuvette in which the emulsion was prepared, and a wide range of ratios of toluene to disperse solvent (up to 1:100 with a total volume of solvents of 840 µL) that were used throughout the investigations. It should be mentioned that the amount of specified volume is enough to combine further measurements with a spectrophotometric detection (using micro-cuvette (about 5 µL) or a cuvetteless spectrophotometer) or chromatographic finishing in case of other analytical applications.

Turbidity indicates the ability of the system to disperse the extraction solvent into fine droplets throughout the aqueous volume of the sample. Therefore, turbidity was measured for all systems (T:M, T:AN, T:A, and T:E) in various volume ratios of 1:1, 1:2, 1:5, 1:10, 1:25, 1:50, and 1:100 (Figure 3) and was correlated with the droplet size of the toluene dispersed in the aqueous phase immediately after mixing. The results of the averaged toluene droplet diameters and surface Sauter diameters at 0 s are presented in Table 1.

**TABLE 1:** Turbidity and average diameters of droplets for various dispersive solvent systems.

Volume ratio	T:M			T:AN			T:A			T:E		
	Turb	d_a_, μm	d_32_, μm	Turb	d_a_, μm	d_32_, μm	Turb	d_a_, μm	d_32_, μm	Turb	d_a_, μm	d_32_, μm
1:1	0.65	63.24	88.62	0.34	62.29	82.90	0.42	56.02	78.40	0.59	54.36	72.22
1:2	1.55	61.44	85.53	0.51	62.36	82.57	0.71	54.62	77.99	1.25	50.01	62.64
1:5	2.71	60.26	78.94	1.23	62.44	81.19	2.03	53.70	76.99	2.26	48.80	60.74
1:10	5.55	54.07	63.70	2.32	58.75	79.13	4.38	48.11	73.73	2.61	48.39	60.06
1:25	7.92	50.97	60.58	6.37	53.21	71.42	12.30	37.60	63.17	23.79	16.25	24.03
1:50	22.85	38.61	48.34	13.16	44.13	56.24	14.33	39.50	66.15	18.60	26.25	33.85
1:100	16.27	44.18	55.52	1.59	58.26	78.11	6.11	49.65	75.46	8.86	41.70	48.26

**FIGURE 4:**
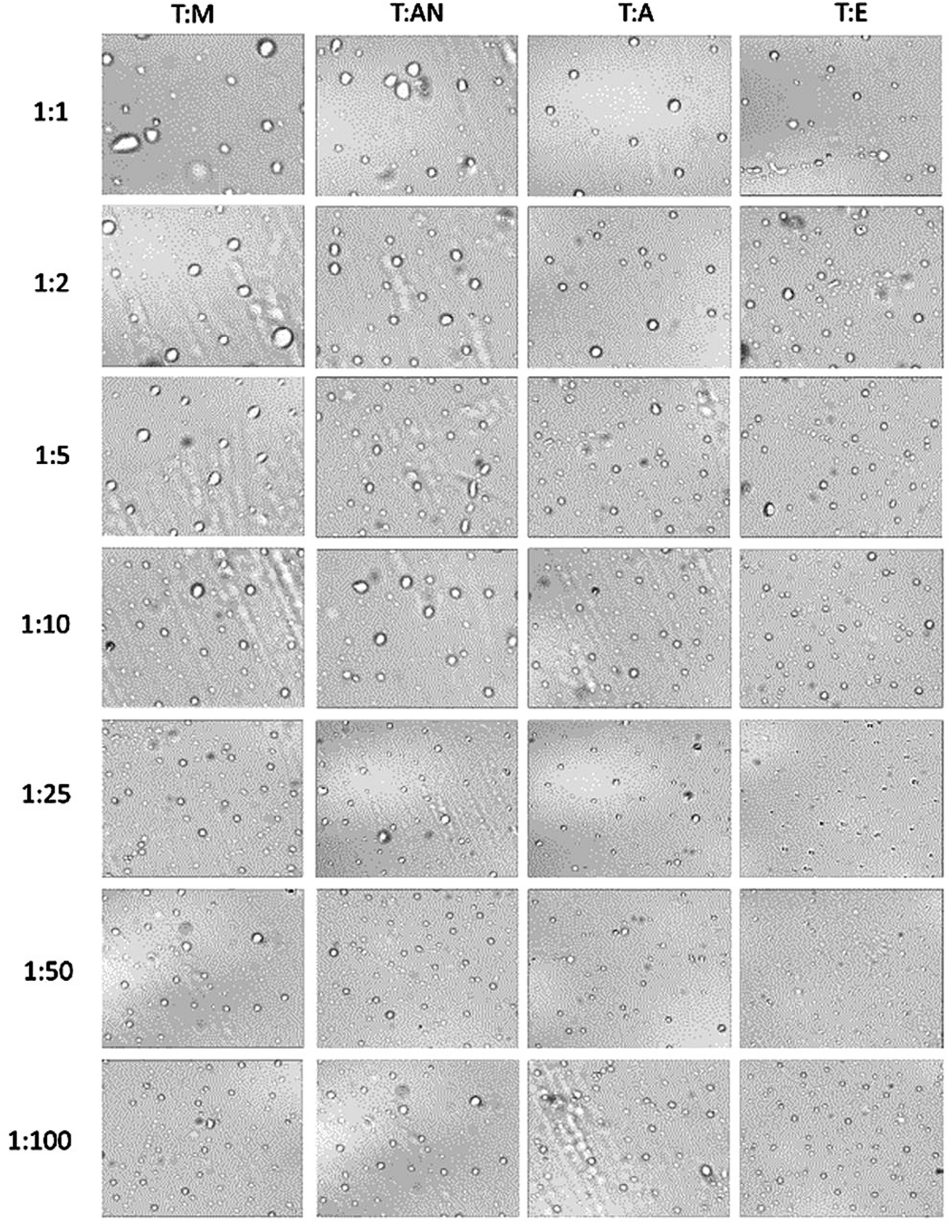
Photomicrographs of T:M, T:AN, T:A, and T:E emulsion particles in 1:1, 1:2, 1:5, 1:10, 1:25, 1:50, and 1:100 volume ratios.

The results indicate that the type of dispersive solvent, as well as its volume, has a strong influence on the formation of emulsions throughout the volume of aqueous samples. The highest turbidity values were obtained for the mixture of toluene and ethanol. In turn, significantly lower values were obtained for acetone and acetonitrile. In all the systems, the turbidity increases as the volume of the dispersive solvent increases, with the highest values of turbidity observed at volume ratios of 1:25 for ethanol and acetone and 1:50 for methanol and acetonitrile. Further increases in volume result in a decrease in turbidity (Figure 3). At ratios of 1:1 to 1:10, the volume of the dispersive solvent is too low to disperse toluene throughout the entire volume of the aqueous solution. On the other hand, at a ratio of 1:100, the volume is too high and greatly affects the polarity of the continuous phase, probably leading to the dissolution of the toluene in water. In the next step, microscopic analyses of the formed emulsions were performed. Examples of the microscopic images are shown in Figure 4, and the exact values for all systems are summarized in Table 1. The results obtained for the size distribution of toluene droplets confirm the results obtained using the turbidimetric method. The smallest average diameters of dispersed extraction solvent were obtained for the toluene:ethanol (1:25) and toluene:methanol (1:50) solvent systems. A demonstration of the formation of a T:E emulsion is shown in the video (See Supplementary Material). Furthermore, a good correlation between droplet size and turbidity was observed, with the determination coefficient (*R* ^2^) ranging from 0.84 to 0.99. This means that the size of dispersed particles in the emulsion can be predicted based on turbidimetric measurements for each system. In the subsequent stage of the study, the stability of the produced emulsions was determined by calculating their half-life. The EHL corresponds to the time it takes for the emulsion volume to decrease to half of its initial volume (Sheng, 2011).

**FIGURE 5:**
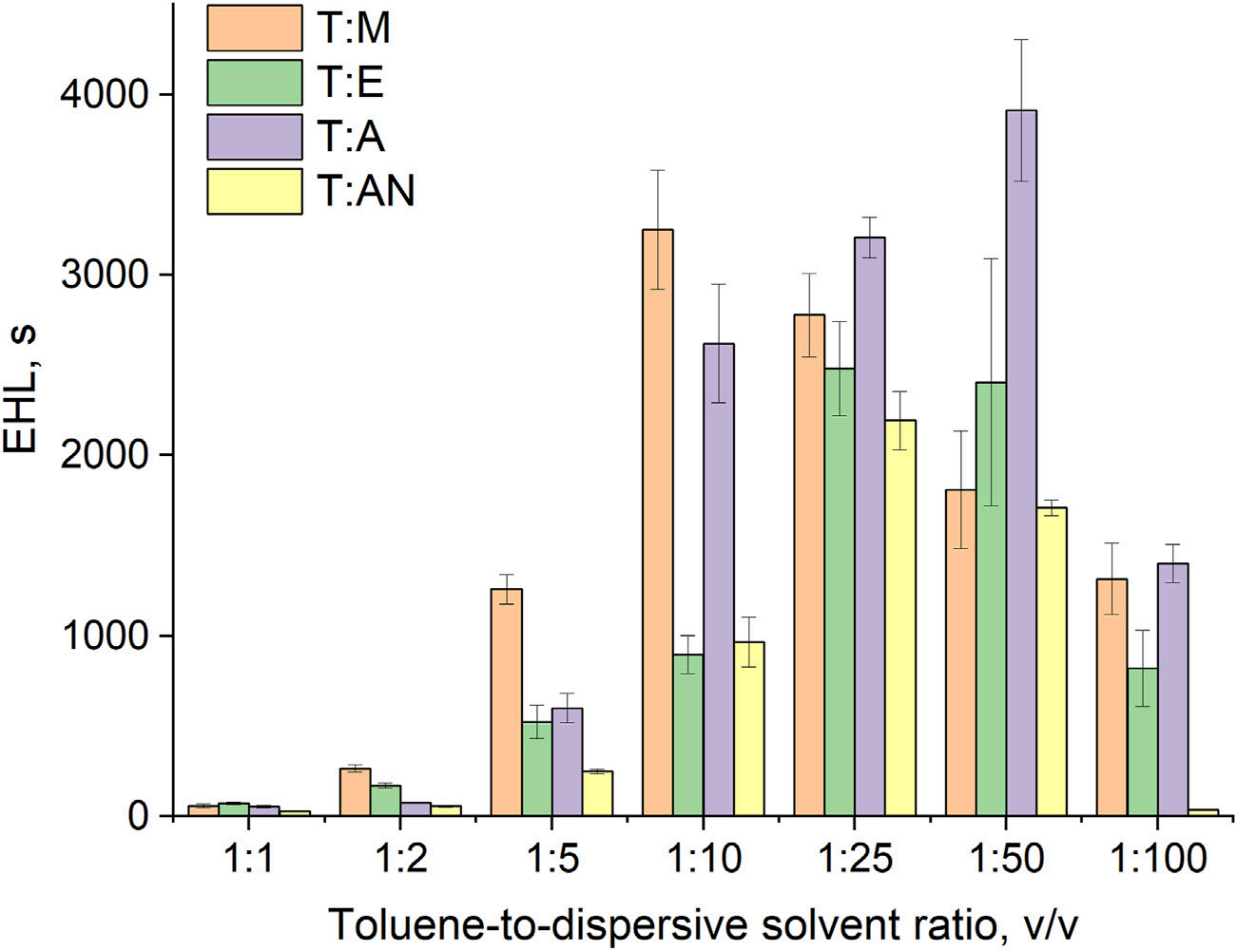
Emulsion half-life of various toluene/dispersive solvent systems.

The EHL results for different emulsion systems are shown in Figure 5, and the exact values are presented in Table 2. These outcomes indicate that the stability of the emulsions depends significantly on the type of dispersive solvent used and its volume ratio. The extraction solvent molecules that form the emulsion tend to spontaneously aggregate into larger masses in an aqueous solution. However, the addition of a dispersive solvent has the effect of reducing the surface tension and stabilizing the emulsion by creating an interfacial barrier that prevents coalescence of the dispersed extraction solvent droplets.

**TABLE 2:** The reaction rate constant (*k*) and emulsion half-life (EHL) for different ratios of toluene:methanol, toluene: acetonitrile, toluene: acetone and toluene: ethanol mixtures.

Volume ratio	T:M		T:AN		T:A		T:E	
	k, s^-1^	EHL, s	k, s^-1^	EHL, s	k, s^-1^	EHL, s	k, s^-1^	EHL, s
1:1	0.01255	55	0.02384	29	0.01306	53	0.00968	72
1:2	0.00272	255	0.01206	57	0.00888	78	0.0041	169
1:5	5.52E-04	1257	0.00278	249	0.00117	592	0.00137	506
1:10	2.14E-04	3232	7.26E-04	954	2.68E-04	2591	8.13E-04	852
1:25	2.51E-04	2766	3.17E-04	2184	2.16E-04	3205	2.82E-04	2461
1:50	3.91E-04	1774	4.06E-04	1709	1.79E-04	3881	3.00E-04	2309
1:100	5.34E-04	1298	0.01942	36	4.57E-04	1515	8.81E-04	787

The greater the reduction in surface tension, the higher the stability of the emulsion (Moldoveanu and David, 2021). Since the dispersive solvents used in this study are completely soluble in water, emulsions with the lowest surface tension exhibited the highest stability and turbidity. For instance, at a ratio of 1:10, the emulsion stability was the highest for T:M and T:A, with values of 3232 and 2591 s, respectively. The surface tension values for methanol and acetone are 22.7 and 25.2 mN/m, respectively. Ethanol theoretically has a low surface tension of 22.1 mN/m, which contributes to the most effective formation of emulsions. However, in the present study, 96% ethanol was used, and the 4% water content in ethanol increased the surface tension value, thereby reducing the stability of the formed emulsion. Similarly, the system containing acetonitrile exhibited low stability due to its surface tension value of 29.04 mN/m.

In each system, an increase in emulsion stability is observed with an increase in the volume of the dispersive solvent. Subsequently, maximum emulsion stability is reached, followed by a gradual decrease upon further increasing the volume of the dispersive solvent. The maximum emulsion stability was obtained at volume ratios of extraction solvent to dispersive solvent of 1:10, 1:25, 1:50 and 1:25 for the T:M, T:AN, T:A and T:E systems, respectively. These results indicate that if too little dispersive solvent is used, it will not be able to break down the continuous phase of the extraction solvent into stable small droplets and lower the surface tension of the system. On the other hand, an excessive amount of dispersant also has an adverse effect, due to a significant decrease in the viscosity of the systems, which leads to a decrease in the stability of the emulsions.

### 3.2 Study of mechanical emulsification

**FIGURE 6:**
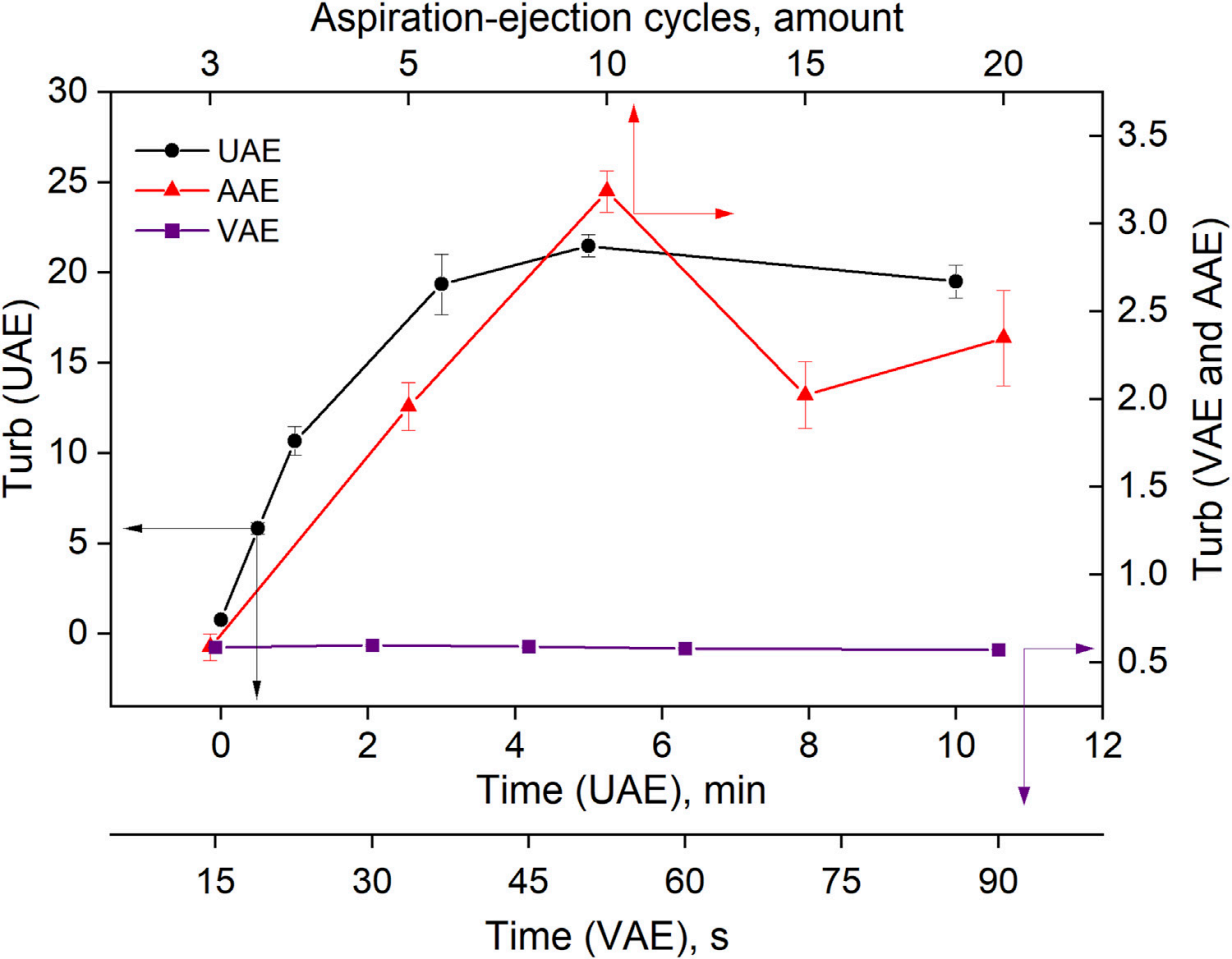
Effect of the variables on the turbidity of the toluene-water system in UAE, VAE, AAE emulsification procedures.

Mechanical emulsification using ultrasound energy, vortex mixing and air mixing was also studied. The dependence of the turbidity of the toluene-water system on sonication time (UAE procedure), vortex mixing time (VAE procedure) and the number of aspiration-injection cycles (AAE procedure) is shown in Figure 6. An increase in the time of ultrasound in the range of 0–3 min led to an increase in the turbidity of the system. After that, the turbidity changed only slightly. The duration of the vortexing (VAE procedure) did not have a large effect on turbidity, and its values remained low throughout the experiment. In the AAE procedure, the highest turbidity was observed after 10 aspiration-injection cycles; it then decreased.

**FIGURE 7:**
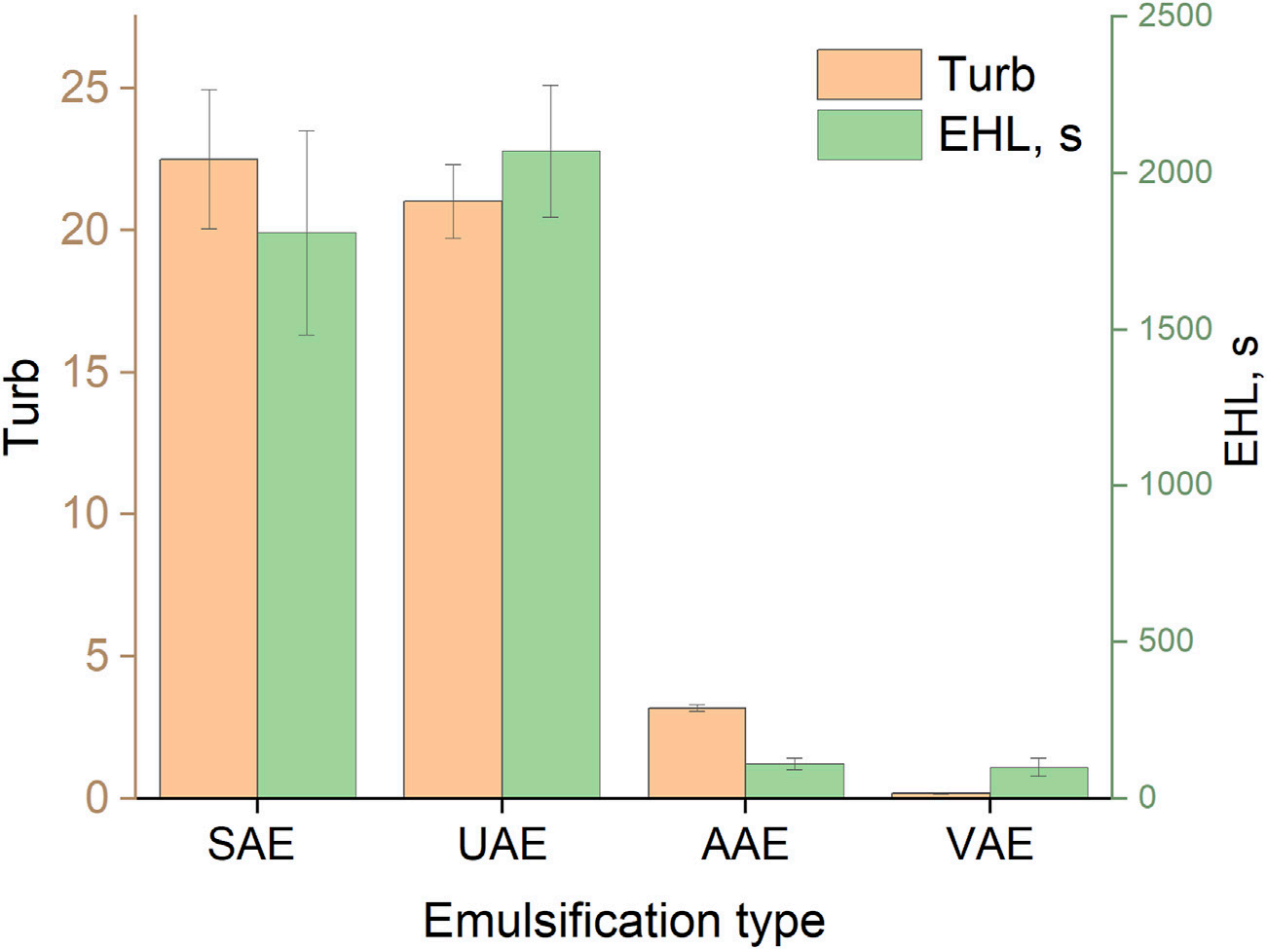
Turbidity and EHL values for different methods of emulsification. SAE: T:M ratio, 1:50; UAE: ultrasonication time, 5 min; VAE: vortexing time, 30 s; AAE: aspiration-injection cycles, 10.

The turbidity values and corresponding EHL values of the emulsion of toluene in water, obtained using methanol as a dispersive solvent (T:M = 1:50) and auxiliary energies, are shown in Figure 7. It can be seen that systems with high turbidity (UAE and SAE) were the most stable among those investigated. This is probably related to the good dispersion of toluene in water caused by the strong effect of ultrasound in the UAE procedure or the influence of the dispersive solvent in the SAE procedure (as discussed above).

### 3.3 Comparison of different types of microextraction for determination of anionic surfactants

**FIGURE 8:**
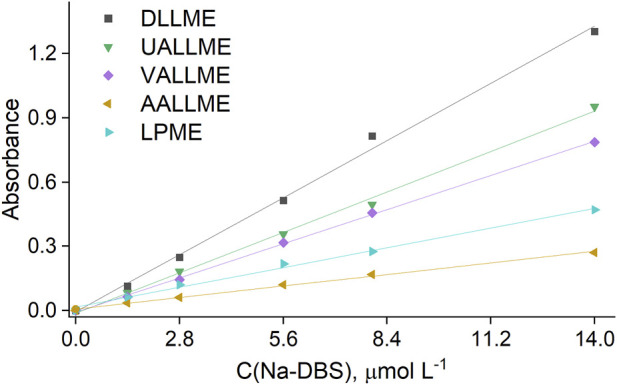
Calibration curves for the determination of anionic surfactants with CV, obtained by different extraction procedures.

The results obtained from the study of cloudy state formation were used to develop microextraction procedures, namely, conventional DLLME, UALLME, VALLME and AALLME. Anionic surfactants were selected as the model analyte, and spectrophotometry was chosen as the detection technique. Although the aim was to observe the experimental conditions used in the kinetic study to as great an extent as possible (see Sections 2.3.1, 2.3.2), in some cases their subtle modification was necessary as a result of specifics of the analytical determination (see Section 2.5). These conditions were chosen based on the maximum extraction efficiency (EF) of the target analyte, despite the fact that the maximum turbidity values of the system found in Sections 3.1, 3.2 were not necessarily achieved. The calibration plots for the various microextraction procedures are shown in Figure 8.

**FIGURE 9:**
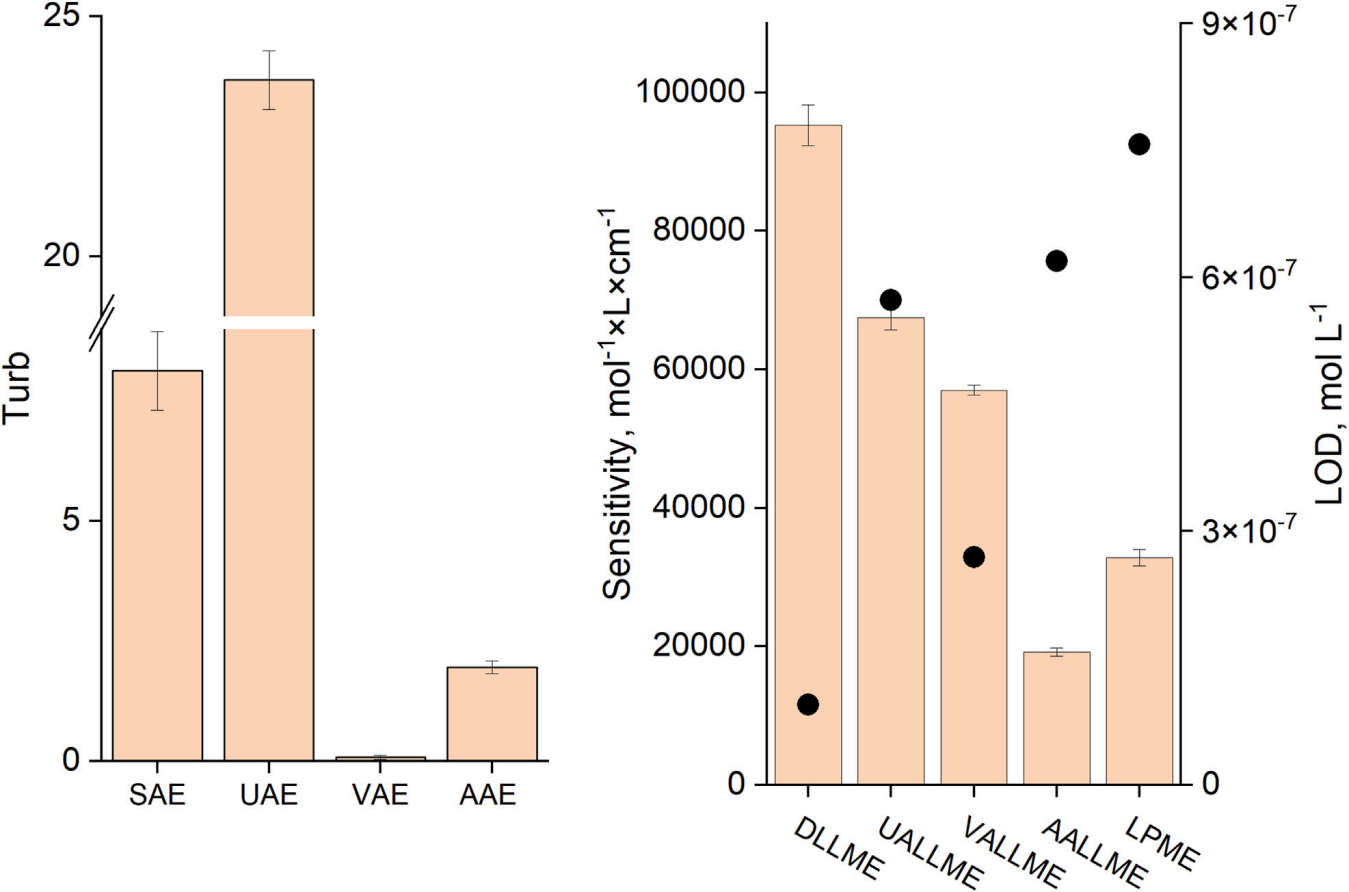
Turbidity values for different methods of emulsification **(A)** and sensitivity **(B)** (slope of the calibration curve and LOD in mol L^-1^, black dots) of the respective analytical procedures. SAE (DLLME): T:M ratio, 1:25; UAE (UALLME): ultrasonication time, 5 min; VAE (VALLME): vortexing time, 60 s; AAE (AALLME): aspiration-injection cycles, 5.

Among the microextraction methods, the conventional DLLME approach was the most sensitive (with EF of 67%) when methanol was used as the dispersive solvent in a 1:25 ratio, despite the fact that the turbidity of this system was not the highest (Figure 9). This could be explained by the rapid and more complete spreading of the formed emulsion throughout the aqueous phase and the positive effect of alcohols (methanol and ethanol) on the extraction efficiency of anionic surfactants with cationic dyes, as previously reported by other authors (Kawase et al., 1979; Kawase and Yamanaka, 1979; Kawase, 1980; Del Valle et al., 1988).

The highest turbidity (near 23) was achieved when ultrasound emulsification was used, but the extraction efficiency (EF of about 48%) was not the best among the studied approaches. This could be explained by the fact that toluene, an extraction solvent lighter than water, forms a thin film on the surface of the aqueous phase, and this needs to be disrupted by shaking to initiate dispersion of the extraction solvent; otherwise, the extraction efficiency and measurement precision could be low. It should be noted that after sonication, the formed emulsion occupied only about half of the upper part of the aqueous phase. As a result, it is difficult to achieve high analyte recoveries.

The lowest turbidity was observed in vortex-assisted emulsification (VAE), but the extraction efficiency of VALLME (EF of about 40%) was second among mechanical emulsification methods (after UALLME). This means that the efficiency of vortex emulsification is low, but extraction of the target analyte is high. This could be explained thus: that despite the weak formation of toluene droplets in the aqueous phase (low turbidity value), the intensive mixing of the aqueous and extraction phases and continuous contact of toluene with the fresh portions of the continuous phase led to a high efficiency of the VALLME process. The sensitivity of the anionic surfactant determination was the worst for the AALLME and LPME methods with EF of 14% and 23% respectively.

### 3.4 Analytical figures of merit

Based on the obtained results, a DLLME method for the determination of anionic surfactants in water samples was developed. In the method, methanol was used as the dispersive solvent. Under the optimized experimental conditions, a calibration plot was constructed from five data points over the range of 1.4–14 × 10^−6^ mol L^−1^ (or 0.49–4.9 mg L^-1^). The regression equation was A = 95,247×*C*–0.006 (where A means the absorbance and *C* is the concentration of DBS in mol L^−1^) with a correlation coefficient of 0.9962, thus proving a good linear relationship between absorbance and DBS concentration. The LOD and LOQ, calculated as three- and ten-times the standard deviation of the blank tests, were found to be 9.5 × 10^−8^ and 3.2 × 10^−7^ mol L^−1^ (or 0.033 and 0.110 mg L^-1^) respectively. The precision and accuracy of the suggested method were checked by performing three extractions of spiked samples at two concentration levels (0.2 and 1.2 × 10^−5^ mol L^−1^) over two consecutive days. The results are shown in Supplementary Table S1 (Supplementary Material).

### 3.5 Analytical application

Water samples were used to check the applicability of the developed method. The results are presented in Supplementary Material, section *“Analytical application”*.

## 4 Conclusion

The emulsification processes that take place during various liquid-phase microextraction procedures were investigated. For chemical dispersion, extraction-to-dispersive solvent ratios of 1:25–1:50 were found to be best in terms of the initial turbidity values. A smaller volume of dispersive solvent is insufficient to disperse the extraction solvent throughout the entire volume of the aqueous sample. Conversely, using too much dispersive solvent (1:100) alters the polarity of the continuous phase and increases the solubility of the extraction solvent. The same parameters have a significant impact on the stability of the formed emulsion. Among the dispersive solvents, ethanol showed the best results, probably due to having the lowest surface tension. In the case of mechanical dispersion, the best results were achieved using ultrasound, and were comparable to chemical dispersing. Both high initial turbidity values (near 23) and long half-lives (2070 s) were observed. The best results were achieved with ultrasonication for 5 min. Based on these findings, a DLLME method was developed for the determination of DBS in water samples. The method has a limit of detection of 0.033 mg L^-1^ and a linear range for the anionic surfactant (as sodium dodecyl benzene sulfonate) from 0.49 to 4.9 mg L^-1^.

To achieve high efficiency in the DLLME extraction process and maximize analyte recovery, several conditions must be fulfilled (however, we must emphasize that all the resulting conclusions apply only to our experimental system and analyte, and it is not possible to transfer them to other systems/analytes without thorough experimental verification):

• The emulsion should be formed throughout the entire sample volume as quickly as possible.
• The extraction solvent should be dispersed into the smallest possible droplets.
• The emulsion should only remain stable for a limited period of time to simplify the centrifugation step.

## Data Availability

The original contributions presented in the study are included in the article/Supplementary Material, further inquiries can be directed to the corresponding authors.

## Abbreviations

AALLME, Air-assisted LLME; DLLME, Dispersive liquid–liquid microextraction; EHL, Emulsion half-life; LLME, Liquid–liquid microextraction; LPME, Liquid-phase microextraction; T: A, Toluene–acetone system; T: AN, Toluene–acetonitrile system; T:E, Toluene–ethanol system; T:M, Toluene–methanol system; UALLME, Ultrasound-assisted LLME; VALLME, Vortex-assisted LLME.
