# Supplementary material for: A closer look at how the dispersive liquid–liquid microextraction method works. Investigation of the effect of solvent mixture composition on the quality and stability of the cloudy state
Source: Front Chem. 2024 Jun 11;12:1383445. doi: 10.3389/fchem.2024.1383445 (PMC11196600; doi:10.3389/fchem.2024.1383445)
Supplement: Supplementary file 2 [file DataSheet1.docx]

**A closer look at how the dispersive liquid–liquid microextraction method works. Investigation of the effect of solvent mixture composition on the quality and stability of the cloudy state**

Serhii Zaruba ^a^, Michaela Ovšonková ^a^, Patrycja Makoś-Chełstowska ^b^, and Vasil Andruch ^a^

*^a^ Department of Analytical Chemistry, Institute of Chemistry, Faculty of Science, Pavol Jozef Šafárik, University in Košice, SK-04154 Košice, Slovak Republic*

*^b^ Department of Process Engineering and Chemical Technology, Faculty of Chemistry, Gdańsk University of Technology, 80-233 Gdańsk, Poland*

**Supplementary material**

**Figure S1** Examples of kinetic curves of emulsion sedimentation (upper images) and their linearized version (lower images).

**Figure S2** Effect of the CV concentration (A), pH (B), concentration of Na_2_SO_4_ (C), toluene to methanol ratio (D).

**Table S1** Intra-day and inter-day precision and accuracy data for the determination of DBS (×10^5^ mol L^−1^)

**Table S2** Determination of anionic surfactants in water samples

**Procedures for microextraction surfactant determination**

*Procedure for* *conventional DLLME determination*

First, 220 μL of methanol containing 8.3 μL of toluene was rapidly injected into the pretreated aqueous sample using a chromatographic syringe. The mixture was then gently mixed by inverting the test tube 3 times to distribute the formed emulsion throughout the whole volume. In this step, the formed ion associate of DBS-CV was extracted into the toluene droplets. The mixture was then centrifuged at 3000 rpm for 5 min for phase separation. The extract was diluted with 300 μL of toluene to increase the total volume, and 250 μL of the obtained phase was withdrawn by automatic pipette, transferred into a 2 mm glass cell and the absorbance was measured at 608 nm.

*Procedure for UALLME determination*

First, 8.3 μL of toluene was carefully placed on the surface of the pretreated aqueous sample using a chromatographic syringe. The mixture was vigorously shaken by hand 20 times to break the thin film of extraction solvent on the surface, as was done during the emulsification study described in Section “*Ultrasound-assisted emulsification (UAE)”*. Then the tube was immediately placed into an ultrasonic water bath and was sonicated for 5 min. After sonication, the mixture was gently mixed 3 times by inverting the test tube to distribute the emulsion throughout the volume. In this step, the formed ion associate of DBS-CV was extracted into the toluene droplets. The procedure was then followed as described in section “*Procedure for conventional DLLME determination” (ESI)*.

*Procedure for VALLME determination*

First, 8.3 μL of toluene was carefully placed on the surface of the pretreated aqueous sample using a chromatographic syringe. The mixture was shaken on the vortex agitator at 3000 rpm for 60 s. In this step, the formed ion associate of DBS-CV was extracted into the toluene phase. The procedure was then followed as described in section “*Procedure for conventional DLLME determination” (ESI)*.

*Procedure for AALLME determination*

First, 8.3 μL of toluene was carefully placed on the surface of the pretreated aqueous sample using a chromatographic syringe. The mixture was then aspirated into a 5 mL PE syringe from the test tube and then pushed out (5 times). A cloudy state was formed, and the formed ion associate of DBS-CV was extracted into the toluene droplets. The procedure was then followed as described in section “*Procedure for conventional DLLME determination” (ESI)*.

*Procedure for LPME determination*

First, 8.3 μL of toluene was carefully placed on the surface of the pretreated aqueous sample using a chromatographic syringe. The tube was being gently shaken for 10 min and the formed ion associate of DBS-CV was extracted into the toluene phase. The procedure was then followed as described in section “*Procedure for conventional DLLME determination” (ESI)*.

**Figure S1** Examples of kinetic curves of emulsion sedimentation (upper images) and their linearized version (lower images).

**Investigation of appropriate experimental conditions for DLLME**

The main experimental conditions for DLLME determination of anionic surfactants were taken from ^16^ checked and adjusted to microextraction conditions. The effect of these conditions on the absorbance extracts is shown in the Fig. S3. Based on the obtained results, the following experimental conditions were chosen: CV concentration 2×10^-4^ mol L^-1^; pH 5.0, concentration of Na_2_SO_4_ 0.4 mol L^-1^, toluene to methanol ratio 1:25.

**Figure S2** Effect of the CV concentration (A), pH (B), concentration of Na_2_SO_4_ (C), toluene to methanol ratio (D).

**Table S1** Intra-day and inter-day precision and accuracy data for the determination of DBS (×10^5^ mol L^−1^)

| DBS added | Intra-day | | | Inter-day | | |
| --- | --- | --- | --- | --- | --- | --- |
|  | Determined | RSD, % | Recovery, % | Determined | RSD, % | Recovery, % |
| 0.200 | 0.192 ± 0.010 | 2.7 | 96.0 | 0.194 ± 0.020 | 3.5 | 97.0 |
| 1.20 | 1.27 ± 0.20 | 6.3 | 106 | 1.29 ± 0.17 | 5.30 | 108 |

**Analytical application**

In real water samples, the concentrations of surfactants were under the LOQ of the proposed method. Thus, the water samples were spiked with a known amount of DBS. As can be seen from Table S2, the added DBS were determined accurately, testifying to the absence of sources of systematic errors in the anionic surfactant determination.

**Table S2** Determination of anionic surfactants in water samples

| Sample | DBS added, mg L^-1^ | DBS found, mg L^-11^ | RSD, % | Recovery, % |
| --- | --- | --- | --- | --- |
| Tap water | 0 | < LOQ | - | - |
|  | 0.49 | 0.48 ± 0.08 | 6.8 | 98.0 |
|  | 0.98 | 1.00 ± 0.04 | 1.5 | 102 |
| Well water | 0 | < LOQ | - | - |
|  | 0.49 | 0.53 ± 0.04 | 3.1 | 108 |
|  | 0.98 | 0.99 ± 0.11 | 4.7 | 101 |
